# Petersen’s Space Hernia: An Uncommon Diagnosis With Potentially Fatal Consequences

**DOI:** 10.7759/cureus.101235

**Published:** 2026-01-10

**Authors:** Rui Bernardino, Pedro D Santos, David Aparício, Luís Miranda

**Affiliations:** 1 General Surgery, Unidade Local de Saúde de Santa Maria, Lisbon, PRT

**Keywords:** acute care surgery and gastrointestinal surgery, bariatric surgery complications, gastric bypass surgery, petersen's hernia, strangulated internal hernia

## Abstract

Petersen’s space hernias are a rare but potentially life-threatening complication following Roux-en-Y gastric bypass, seen in both bariatric and oncologic contexts. Although evidence is mixed, some studies suggest a higher risk of internal herniation after laparoscopic procedures. A 52-year-old male patient with a prior history of a minimally invasive bariatric Roux-en-Y gastric bypass presented with acute left upper quadrant pain and nausea. Imaging revealed small bowel obstruction with mesenteric twisting, consistent with an internal hernia. Exploratory laparotomy confirmed a Petersen’s space hernia, with nearly the entire small intestine herniated beneath the alimentary limb and associated large bowel rotation. The bowel was viable after reduction, and the Petersen defect was closed using delayed-absorbable sutures. The postoperative course was uneventful, and the patient was discharged on day four with full recovery. This case underscores the diagnostic difficulty and urgency of surgical management in Petersen’s space hernias, highlighting the importance of early recognition to prevent bowel ischemia and fatal outcomes.

## Introduction

Petersen’s space hernias are internal hernias in which small bowel herniates through the potential space between the mesentery of the Roux limb and the transverse mesocolon after Roux-en-Y reconstruction. This is a recognized late complication that can cause bowel obstruction and ischemia and happens on procedures performed for both bariatric and oncologic indications [1-3]. With the global rise in obesity, the number of patients undergoing bariatric procedures such as gastric bypass has increased substantially [4,5]. Current literature reports an incidence between 0.5% and 5% for this complication after Roux-en-Y gastric bypass [3,6]. Even though data is equivocal, some evidence suggests that laparoscopic procedures have an increased risk of post-op internal hernias [6,7].

Although uncommon, Petersen’s space hernias represent a diagnostic challenge and require prompt surgical intervention to prevent catastrophic outcomes such as intestinal ischemia, postoperative short bowel syndrome, and potentially death.

This paper reports a case of successful surgical resolution of a Petersen's space hernia and highlights the diagnostic challenges associated with this uncommon post-op complication.

## Case presentation

A 52-year-old man presented to the emergency department with a 24-hour history of abdominal pain, predominantly localized to the left upper quadrant, accompanied by nausea but no vomiting. Bowel habits remained unchanged, and the patient denied fever, gastrointestinal bleeding, or other systemic symptoms.

On examination, the patient appeared distressed and maintained a fetal position as an antalgic posture. Vital signs were within normal limits: blood pressure 120/70 mmHg, heart rate 80 bpm, and temperature 37.1 °C. Abdominal inspection revealed multiple small scars consistent with a prior laparoscopic Roux-en-Y gastric bypass performed two years earlier for the treatment of obesity. Palpation elicited tenderness in the left upper quadrant, without signs of peritoneal irritation. The remainder of the physical examination was unremarkable.

Diagnostic assessment and therapeutic intervention

Initial investigations included an abdominal X-ray and routine laboratory tests.

**Figure 1:**
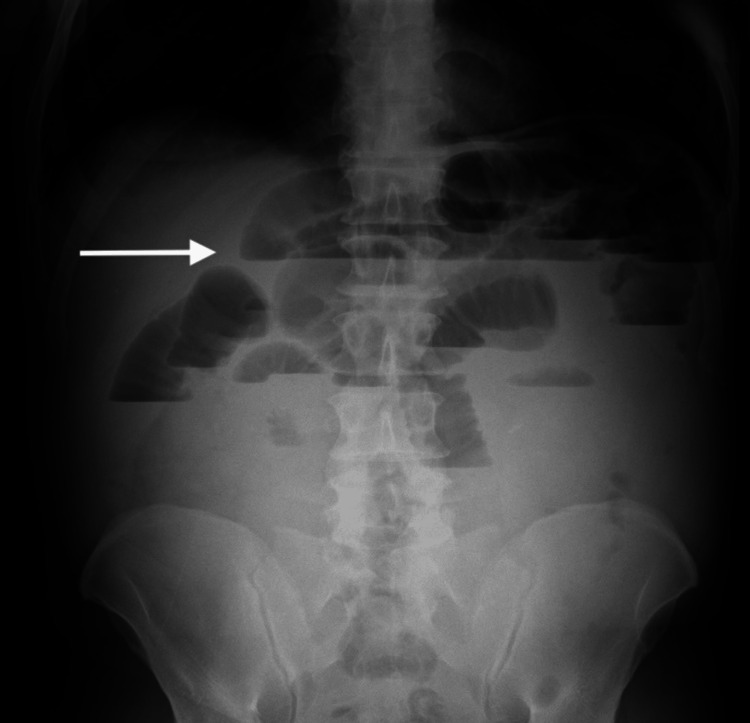
Preoperative abdominal X-ray The arrow points to air-fluid levels on distended bowel loops consistent with small bowel obstruction

The abdominal X-ray (Figure 1) revealed distended small bowel loops with multiple air-fluid levels (arrow) distributed throughout the abdomen, consistent with small bowel obstruction; no pneumoperitoneum was identified. Laboratory results showed no significant changes (Table 1).

**Table 1:** Laboratory results

Lab test	Result	Reference range
Hemoglobin	14.8 g/dL	13.0 – 17.5 g/dL
Hematocrit	44%	40.0 – 50.0%
Leucocytes	10.5 (x10^9)/L	4.0 – 11.0 (x10^9^)
Platelets	182 (x10^9)	150 – 450 (x10^9^)
INR	1.07	0.8 – 1.2
Glicose	132 mg/dL	70 – 110 mg/dL
Creatinine	0.93 mg/dL	0.7 – 1.2 mg/dL
Urea	36 mg/dL	16 – 49 mg/dL
Sodium	133 mmol/L	135 – 145 mmol/L
Potassium	4.2 mmol/L	3.5 – 5.1mmol/L
Alanine transaminase	15 U/L	0 – 41 U/L
Aspartate aminotransferase	25 U/L	0 – 40 U/L
Bilirubin (total)	1.09 mg/dL	< 1.2 mg/dL
Lactate dehydrogenase	237 U/L	100 – 250 U/L

**Figure 2:**
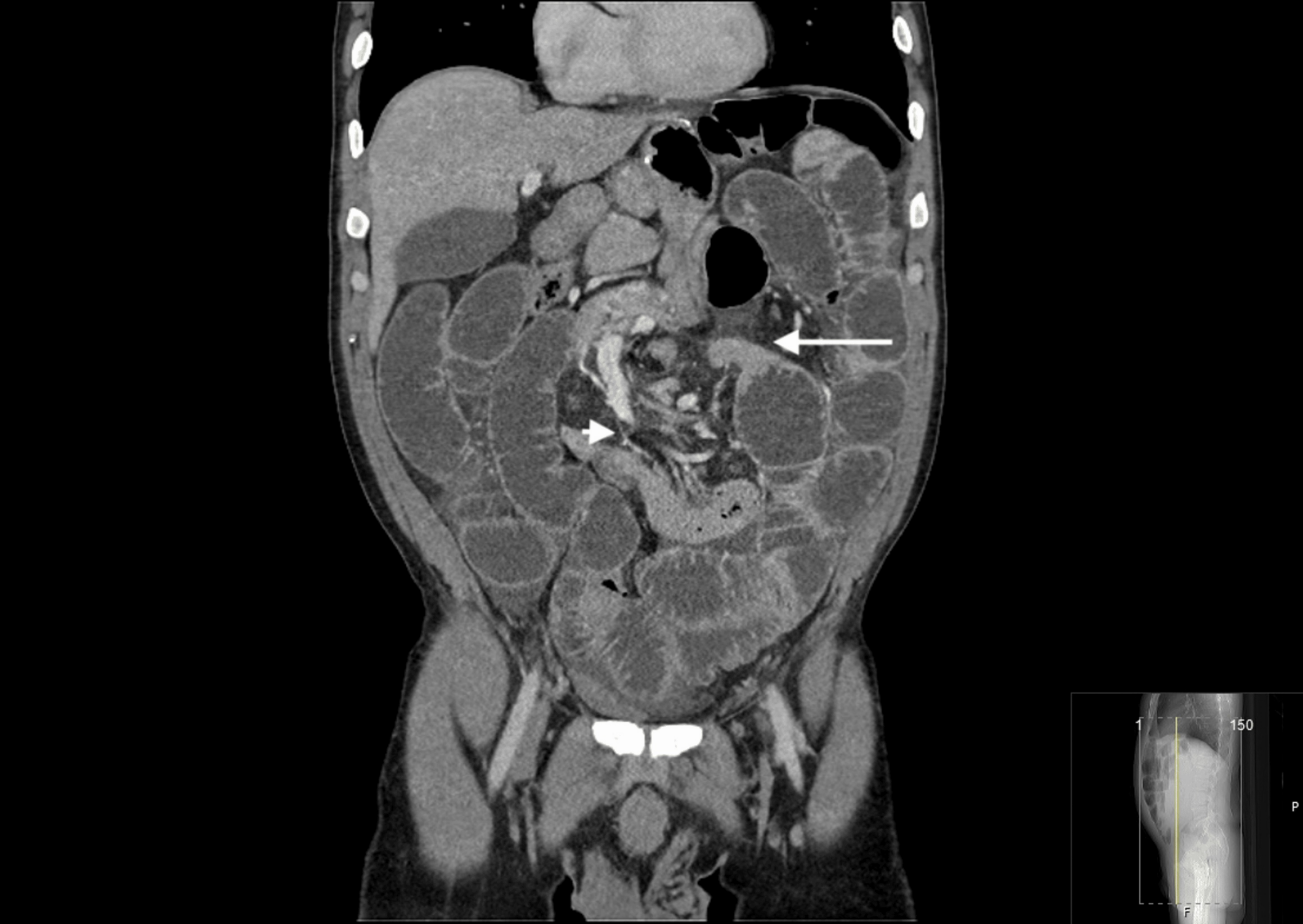
Preoperatory CT scan Short arrow: Mesenteric twisting; Long arrow: Distended bowel loops with a transition point close to the root of the mesenterium

Given these findings, an abdominopelvic CT scan was performed (Figure 2), which demonstrated distended bowel loops passing under the antecolic alimentary limb of the Roux-en-Y bypass with mesenteric twisting (short arrow) with a transition point close to the root of the mesenterium (long arrow), findings highly suggestive of an internal hernia.

**Figure 3:**
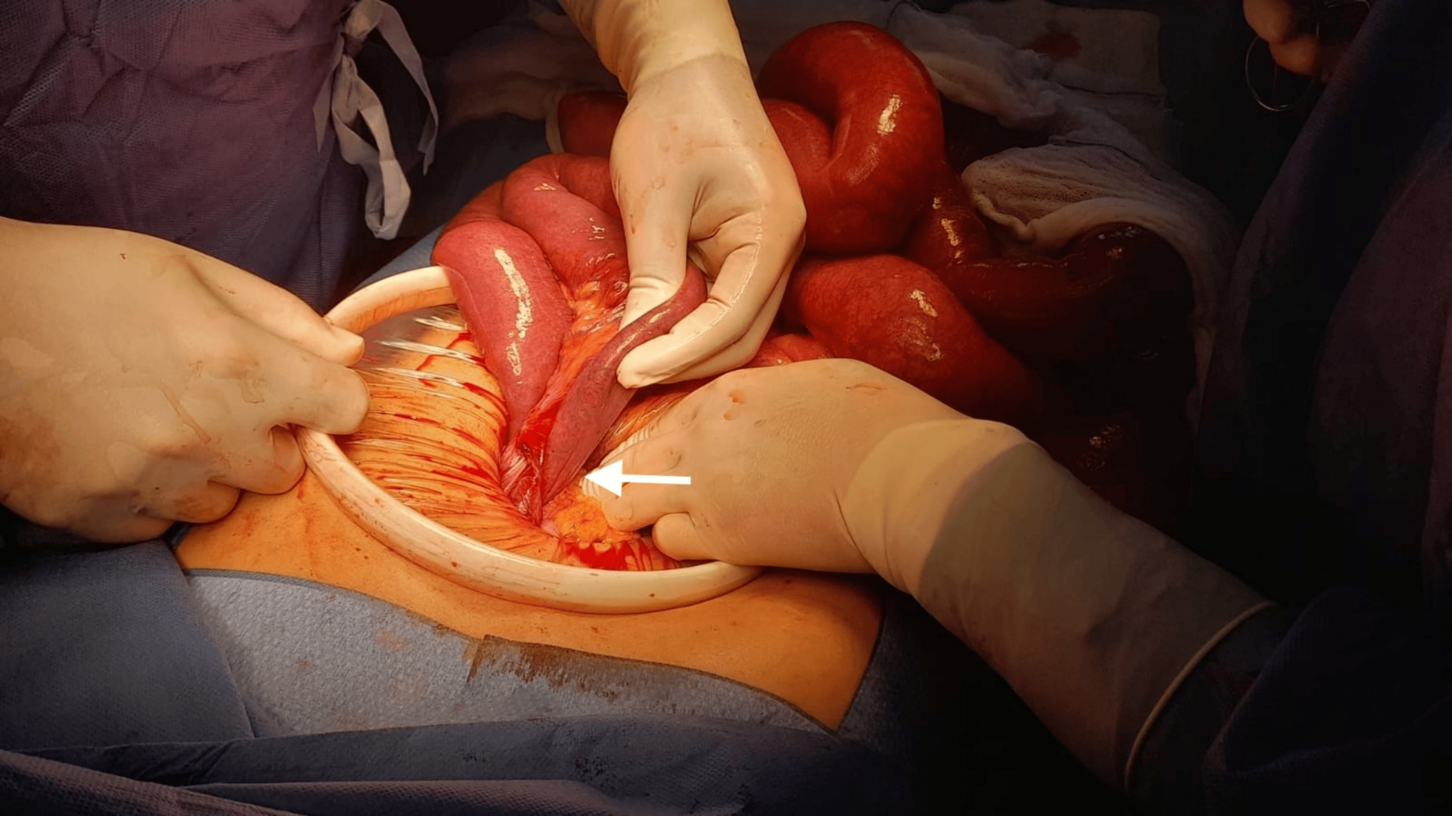
Intraoperative findings Arrow: Petersen’s space hernia involving nearly the entire small intestine herniated beneath the alimentary limb of the bypass

Due to the significant bowel distension, the surgical team elected to proceed with an exploratory laparotomy rather than a laparoscopic approach. Intraoperative findings (Figure 3) confirmed a Petersen’s space hernia involving nearly the entire small intestine herniated beneath the alimentary limb of the bypass (arrow). The large bowel was also rotated, with the cecum displaced to the upper left quadrant.

The small bowel showed venous congestion but remained viable. Gentle reduction of the herniated loops was performed, followed by inspection of both the gastrojejunostomy and jejunojejunostomy to exclude additional complications. Petersen’s space was closed using interrupted delayed-absorbable sutures (PDS, Ethicon) consistent with our institution current practice. The abdominal wall was closed in layers with continuous delayed-absorbable material.

Outcome and follow-up

**Figure 4:**
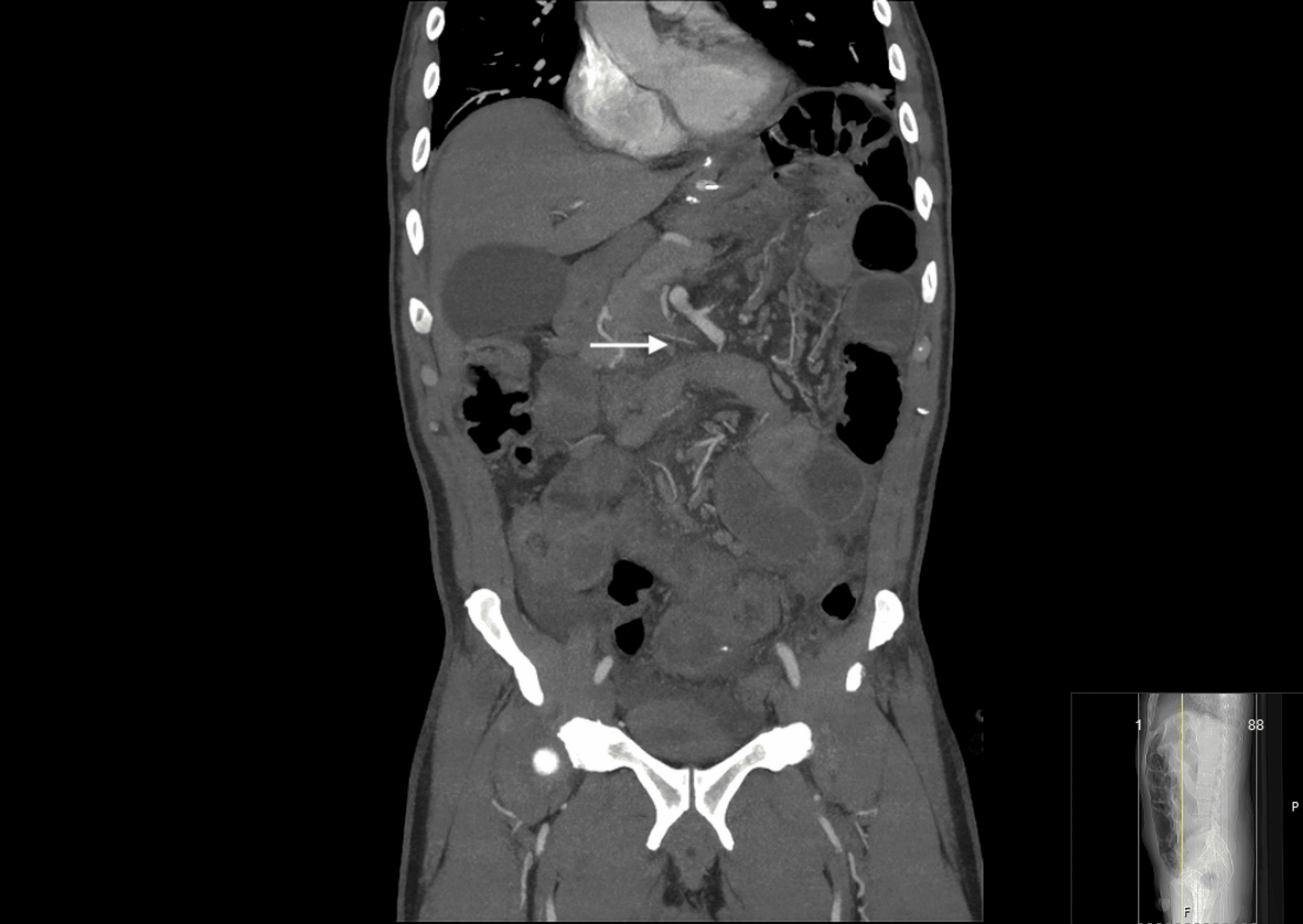
Postoperative CT scan Arrow: Resolution of the mesenteric rotation

Postoperatively, the patient experienced moderate abdominal pain requiring epidural analgesia during the first two postoperative days. Only prophylactic antibiotic therapy was prescribed with two doses of cefoxitin (in accordance with hospital and national guidelines) being administered intraoperatively. A control CT scan (Figure 4) on postoperative day 2 showed no abnormal findings with resolution of the mesenterial rotation (arrow) and consistent small bowel size along its length. The patient was started on a clear liquid diet on postoperative day 2 and gradually advanced to a regular diet during the following 48h. The patient was discharged on postoperative day 4 with well-controlled pain, adequate oral intake, and normal bowel function. The patient attended a follow-up consultation two weeks after surgery, during which no complications were identified with the patient reporting normal oral intake and no complaints. Two teleconsultations were subsequently conducted, confirming an uneventful postoperative course. The patient returned to his home country where further follow-up will be continued by the bariatric team responsible for the initial procedure.

## Discussion

Although rare, Petersen’s space hernias are a serious late complication of Roux-en-Y reconstruction that can lead to bowel obstruction and ischemia. The condition has gained increasing attention due to its association with significant morbidity, particularly when diagnosis is delayed [8]. Early recognition remains challenging, as both clinical and imaging findings are often nonspecific [9].

The incidence of Petersen's hernias after Roux-en-Y gastric bypass varies considerably across institutions and is influenced by surgical technique and follow-up duration. A retrospective observational cohort study of 46918 patients who underwent laparoscopic Roux-en-Y gastric bypass found a cumulative incidence of internal hernia repair of 4.8% at three years of follow-up [10]. However, hernia rates can vary from as low as 0.4% in centers with systematized closure strategies to as high as 8.9% in series without routine mesenteric defect closure [11,12]. A recent five-year single-center study reported a symptomatic Petersen's hernia incidence of 0.4%, with median presentation at 28.1 months post-op and a strong female predominance (90.9%) among operated cases [11].

Multiple risk factors have been identified for internal herniation after Roux-en-Y gastric bypass. Systematic reviews identify non-closure of mesenteric defects, retrocolic limb routing, and laparoscopic approach as predictors for increased risk of internal hernia [3,6]. The role of mesenteric defect closure remains a subject of debate, even though some meta-analyses generally support that routine closure reduces the occurrence of internal hernia [13]. One large institutional series demonstrated a reduction in early internal hernia risk from 8.9% to 2.5% after the implementation of a standardized non-absorbable double-layer suture closure [12].

Petersen's hernia clinical presentation can range from vague intermittent abdominal pain to cases of acute obstruction and strangulation. Reviews and case series emphasize the variability and intermittent nature of the symptoms, including abdominal pain, nausea, and vomiting [6,11]. This variability reinforces the need to maintain a high level of suspicion in patients with a history of gastric bypass presenting with abdominal complaints.

Computed tomography is the preferred preoperative imaging modality, with the mesenteric swirl sign and other indicators of midgut volvulus being the most significant imaging clues that increase the diagnostic suspicion [9,11]. However, CT sensitivity is imperfect, and normal studies do not exclude Petersen's hernia [9,10]. Given the potentially catastrophic consequences of delayed diagnosis, a diagnostic laparoscopy should be performed when clinical suspicion persists despite uncertain imaging findings [7,11] as prompt surgical exploration remains the cornerstone of management to prevent irreversible intestinal damage and reduce mortality risk [8].

The recommended technique is a laparoscopic reduction with closure of Petersen's and other mesenteric defects using interrupted non-absorbable sutures [7]. In the current case report, these steps were performed; however, the defect was closed with a delayed-absorbable suture as is consistent with our institution's current practice for both bariatric and oncological procedures. Even though some literature may favor non-absorbable sutures, the evidence is scarce, and the results are comparable. In cases of acute strangulation, resection of nonviable bowel may be required, and open conversion remains an option depending on patient stability and technical factors [7,12]. Intraoperative indocyanine green angiography may be utilized to assess bowel perfusion before doing any bowel resection [7].

Complication rates vary across series, with bowel necrosis representing the most serious concern. While some center series report no bowel necrosis or mortality in operated Petersen's cases when diagnosed and treated promptly [11], delayed diagnosis can lead to catastrophic outcomes, including short bowel syndrome and death [8]. When treated early via laparoscopy without resection, patients typically experience uneventful recoveries with low recurrence rates when defects are adequately closed [11,12].

The postoperative recovery in our case, with the patient being discharged on the fourth post-operative day, is consistent with outcomes reported in the literature for timely intervention without bowel compromise [14,15]. Even though a laparoscopic approach was first considered, the severity of the bowel distention deemed the minimally invasive approach too risky in our view.

## Conclusions

In emergency care units, a high index of suspicion for this diagnosis should be maintained in patients with a history of Roux-en-Y gastric bypass who present with vomiting, oral intake intolerance, abdominal distension, and/or abdominal pain.

With the increase of bariatric procedures in general with Roux-en-Y gastric bypass being one of the most frequent surgery types for this indication, these complications may become more prevalent in the future. This case underscores the diagnostic difficulty and urgency of surgical management in Petersen's hernias, highlighting the importance of early recognition to prevent bowel ischemia and fatal outcomes. Understanding the pathophysiology, imaging features, and operative management of Petersen’s space hernias is essential for improving patient outcomes in the era of minimally invasive surgery.
